# Tenacious Endemic Typhoid Fever in Samoa

**DOI:** 10.1093/cid/ciaa314

**Published:** 2020-07-29

**Authors:** Michael J Sikorski, Sachin N Desai, Siaosi Tupua, Robert E Thomsen, Jane Han, Savitra Rambocus, Susana Nimarota-Brown, Linatupu Punimata, Salesa Tusitala, Michelle Sialeipata, Seth A Hoffman, J Kathleen Tracy, Ellen E Higginson, Sharon M Tennant, Jillian S Gauld, Daniel J Klein, Susan A Ballard, Roy M Robins-Browne, Gordon Dougan, Eric J Nilles, Benjamin P Howden, John A Crump, Take K Naseri, Myron M Levine

**Affiliations:** 1 Center for Vaccine Development and Global Health, University of Maryland School of Medicine, Baltimore, Maryland, USA; 2 Ministry of Health, Government of Samoa, Apia, Samoa; 3 Microbiological Diagnostic Unit Public Health Laboratory, Department of Microbiology and Immunology, The University of Melbourne at the Peter Doherty Institute for Infection and Immunity, Melbourne, Victoria, Australia; 4 Clinical Translational Research and Informatics Center, Department of Epidemiology and Public Health, University of Maryland School of Medicine, Baltimore, Maryland, USA; 5 Institute for Disease Modeling, Bellevue, Washington, USA; 6 Department of Medicine, University of Cambridge, Cambridge, United Kingdom; 7 World Health Organization, Division of Pacific Technical Support, Suva, Fiji; 8 Centre for International Health, University of Otago, Dunedin, New Zealand

**Keywords:** typhoid fever, epidemiology, Samoa, Oceania, *Salmonella* Typhi

## Abstract

**Background:**

Typhoid fever has been endemic on the island nation of Samoa (2016 population, 195 979) since the 1960s and has persisted through 2019, despite economic development and improvements in water supply and sanitation.

**Methods:**

*Salmonella enterica* serovar Typhi isolates from the 2 hospitals with blood culture capability and matched patient demographic and clinical data from January 2008 through December 2019 were analyzed. Denominators to calculate incidence by island, region, and district came from 2011 and 2016 censuses and from 2017–2019 projections from Samoa’s Bureau of Statistics. Data were analyzed to describe typhoid case burden and incidence from 2008 to 2019 by time, place, and person.

**Results:**

In sum, 53–193 blood culture-confirmed typhoid cases occurred annually from 2008 to 2019, without apparent seasonality. Typhoid incidence was low among children age < 48 months (17.6–27.8/10^5^), rose progressively in ages 5–9 years (54.0/10^5^), 10–19 years (60.7–63.4/10^5^), and 20–34 years (61.0–79.3/10^5^), and then tapered off; 93.6% of cases occurred among Samoans < 50 years of age. Most typhoid cases and the highest incidence occurred in Northwest Upolu, but Apia Urban Area (served by treated water supplies) also exhibited moderate incidence. The proportion of cases from short-cycle versus long-cycle transmission is unknown. Samoan *S*. Typhi are pansusceptible to traditional first-line antibiotics. Nevertheless, enhanced surveillance in 2019 detected 4 (2.9%) deaths among 140 cases.

**Conclusions:**

Typhoid has been endemic in Samoa in the period 2008–2019. Interventions, including mass vaccination with a Vi-conjugate vaccine coadministered with measles vaccine are planned.

Each decade the World Health Organization (WHO) publishes an update on typhoid vaccines that identifies global regions where high disease burdens constitute notable public health problems. In contrast with earlier reports [1, 2], the 2018 update for the first time specifically mentions that typhoid fever is endemic in many island nations of Oceania [3], which includes Polynesia, Micronesia, and Melanesia. Typhoid fever has been endemic in the island nation of Samoa, which has 2 populated islands, Upolu and Savaii (2016 population 152 419 and 43 560, respectively), since at least the 1960s [4, 5].

Branko Cvjetanović, Chief Medical Officer of the Department of Bacterial Diseases at WHO and the most accomplished typhoid epidemiologist of his era (~1950s–1970s), pioneered the first computer-based model of endemic typhoid to predict the impact of providing improved sanitation in a typhoid-endemic area versus mass vaccination with an effective typhoid vaccine, versus both interventions [4]. Cvjetanović used demographic data, endemic typhoid incidence rates, and sanitation data from Samoa in the 1960s, when the country was known as Western Samoa, to populate his computer model. He wrote, “In order to illustrate these uses of the model with an example, we shall take actual data on the epidemiological situation in a small, typical Pacific island with an initial population of about 150 000. The annual birth rate was taken as 35 per 1000 inhabitants, and the annual death rate as 8 per 1000 population. The annual incidence of typhoid fever was taken at the level of 7.2 per 10 000 inhabitants. These data correspond closely to the actual situation in Western Samoa and resembles that in some other islands” [4]. One of the other islands Cvjetanović was referring to was Tonga, where he and colleagues had undertaken a field trial of the effectiveness of acetone-inactivated whole cell typhoid vaccine [6], a vaccine that had shown good protection for up to 7 years in field trials in other sites [7–10]. At an international meeting on the impact of improvements in water supply and sanitation in developing countries in 1981, Cvjetanović commented that the government of Western Samoa wanted to develop tourism as an economic engine but recognized that endemic typhoid was a barrier to achieving that goal, and so they wished to diminish their typhoid burden [5].

The Samoan Ministry of Health and the WHO invited an external consultant, Professor Myron M. Levine, to visit Samoa in early 2013 to review the epidemiologic situation on typhoid endemicity in conjunction with Leausa Toleafoa Dr Take Naseri, the Head of Surveillance, and to propose possible future interventions. One recommendation was to strengthen the clinical microbiology infrastructure in Samoa and to enhance the capacity to perform epidemiologic investigations of typhoid cases. This would better quantify the burden of disease, reveal geographic hotspots, and possibly identify modes of transmission. In 2018 the Bill and Melinda Gates Foundation provided a grant to address that recommendation by supporting the establishment of a Samoa Typhoid Fever Surveillance Initiative (STFSI). Herein the occurrence of blood culture-confirmed typhoid cases during the period January 2008 through December 2019 are presented by time, person, and place to allow better appreciation of the persisting burden of endemic typhoid in Samoa.

## METHODS

A case of typhoid fever was defined as a patient seeking healthcare from whom a blood culture was obtained and *Salmonella enterica* serovar Typhi (*S*. Typhi) was isolated. For the years 2008–2012, blood culture results were available from summaries prepared by the Surveillance Department for the WHO consultancy in March 2013. Blood culture-confirmed cases from 2013 to 2017 were summarized by review of available laboratory records in microbiology laboratories of Tupua Tamasese Meaole (TTM) Hospital on Upolu and Malietoa Tanumafili II (MT2) Hospital on Savaii. Clinical charts related to each case were retrospectively reviewed to collect relevant demographic and clinical information. Data from 2018 and 2019, during which time the STFSI was in place (Q4 2018 and all of 2019), represent prospectively collected and collated data using the Research Electronic Data Capture (REDCap) data management system tailored to the STFSI [11, 12].

During the study period, various bacteriologic methods were employed to confirm the identity of Gram-negative bacilli growing in blood cultures. Broth from blood culture bottles yielding growth was subcultured on blood agar and either MacConkey, xylose-lysine-desoxycholate, or *Salmonella-Shigella* agar plates, as available. Suspicious colonies were inoculated into triple-sugar-iron (TSI) agar slants. Growth from TSI slants showing typical *S*. Typhi biochemical reactions were either agglutinated with specific antisera (anti-Vi, anti-*Salmonella* group D, and anti-*Salmonella* phase 1 flagella H[d]) or inoculated into an API 20E strip (bioMérieux) to confirm *S*. Typhi serovar. Sensitivity of *S*. Typhi to relevant antibiotics (ciprofloxacin, trimethoprim/sulfamethoxazole, ampicillin, chloramphenicol, ceftriaxone) was measured by disk diffusion [13–15]. Isolates from 2018 and 2019 were sent to the Microbiological Diagnostic Unit Public Health Laboratory, University of Melbourne, for reference laboratory confirmation and whole genome sequencing. Figures and maps with incidence were created using case residence and census data [16]. No adjustments were made for healthcare seeking behavior, blood culture practices at healthcare facilities, or the days and hours that healthcare facilities are accessible.

## RESULTS

**Figure 1.**
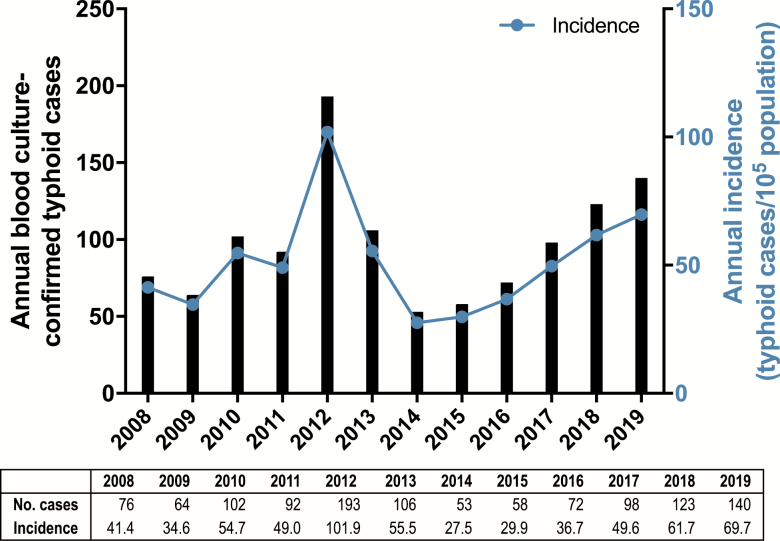
Left: Annual number of blood culture-confirmed typhoid fever cases in Samoa from 2008 to 2019. Right: Annual incidence (blood culture-confirmed cases per 10^5^ persons per year) from 2008 to 2019.

**Figure 2.**
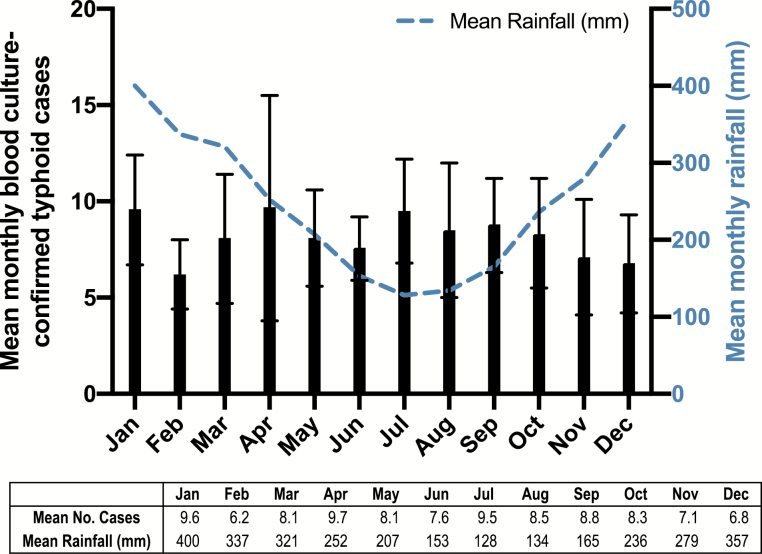
Left: Mean number of blood culture-confirmed typhoid fever cases per month based on aggregated data from 2008 through 2019; error bars represent the 95% confidence interval. Right: Mean monthly precipitation in Samoa from 1909 to 2009 (Source: The World Bank Climate Knowledge Portal).

Figure 1 displays the annual number of blood culture-confirmed cases of typhoid fever in Samoa from 2008 to 2019 (N = 1177). From 2013 onward (N = 650), sex (293 [45.1%] females, 357 [54.9%] males) and age (median = 21 years) were available for every case. Data include cases from both Upolu and the less populated island of Savaii (where blood culture capability was intermittent). When available, culture-confirmed Savaii cases were included. Other than 2012 (193 cases), which included a typhoid outbreak in the prison on Upolu, 53–140 cases annually were confirmed. Figure 1 demonstrates crude annual incidence, which exceeded 40 cases/10^5^ population during 8 of the 12 years. Although Samoa exhibits rainy (November through April, when rainfall is ≥ 250 mm monthly) and nonrainy seasons, there was no apparent evidence of seasonality during the study period (Figure 2).

**Figure 3.**
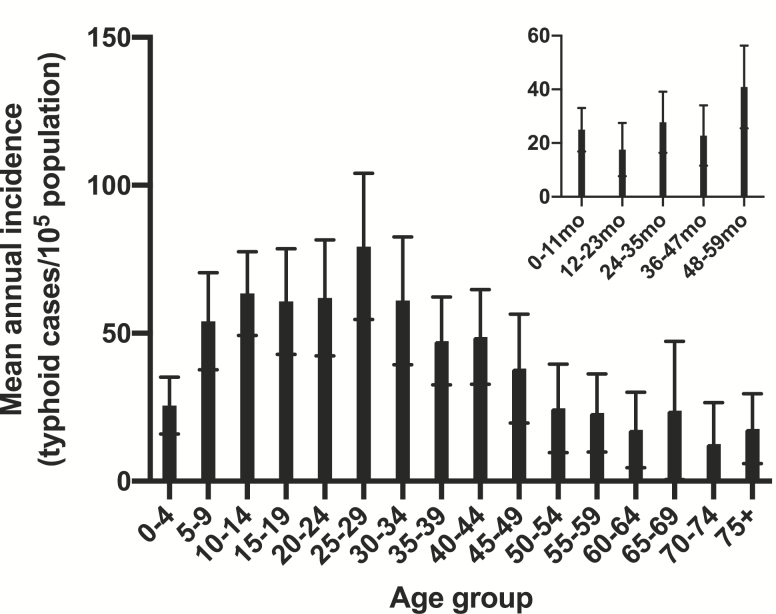
Mean annual incidence of typhoid fever by 5-year age groups, reported as blood culture-confirmed cases per 10^5^ persons per year in Samoa from 2008 to 2019. Insert: The same data from 2013 to 2019 when single-age resolution was available are presented to show incidence by each year of age for the first 5 years of life. Error bars represent the 95% confidence interval.

Typhoid cases were aggregated during the study period to review age-specific incidence rates (Figure 3). The mean incidence in infants < 12 months of age was low (25.0/10^5^ infants) (Figure 3 insert) and remained low (<40/10^5^) through 47 months of age. The incidence increased among preschool children aged 48–59 months (40.9/10^5^) and among school-age children and young adults 5–24 years of age (54.0–63.4/10^5^), and peaked in the 25–29 years age group (79.3/10^5^), before descending incrementally with increasing age (Figure 3). Sex-specific incidence rates were compared for adults in the high-incidence age range of 25–39 years for the 7-year period of 2013–2019. The slightly higher mean annual incidence of confirmed typhoid among males in this age group (70.8/10^5^) was not significantly different from the incidence in females (53.9/10^5^) (*P* = .62, χ ^2^ test, 2-tail, with Yates correction).

Among Samoans > 50 years of age, the incidence dropped rapidly to a level resembling that observed in young children < 48 months of age. The distribution of confirmed typhoid cases by 5-year age groups is shown in Table 1, along with the population of the corresponding age group (2019 population used as reference). Notably, 93.6% of all cases occurred in persons < 50 years of age; the 31 052 Samoans ≥ 50 years of age (15.5% of the total population) collectively contributed only ~6.4% of all cases.

**Table 1.** Total Cases of Blood Culture-confirmed Typhoid Fever in Samoa from 2008 to 2019, the Percentage of Cases by 5-Year Age Groups, and the Cumulative Percentage of Typhoid Cases by Age as a Percentage of the Aggregate Total Number of Cases

Age Group (years)	Population (2019 estimate)^a^	Total cases^b^ (2008–2019)	Percentage of Cases by Age Group	Cumulative Percentage of Typhoid Cases by Age
**0–4**	28 883	83	7.5	7.5
**5–9**	25 663	156	14.0	21.5
**10–14**	21 990	170	15.3	36.7
**15–19**	19 440	141	12.7	49.4
**20–24**	16 329	114	10.2	59.7
**25–29**	13 675	125	11.2	70.9
**30–34**	12 223	87	7.8	78.7
**35–39**	11 077	63	5.7	84.4
**40–44**	10 516	61	5.5	89.8
**45–49**	10 026	42	3.8	93.6
**50–54**	8665	24	2.2	95.8
**55–59**	7190	16	1.4	97.2
**60–64**	5359	10	0.9	98.1
**65–69**	3564	10	0.9	99.0
**70–74**	2757	4	0.4	99.4
**75+**	3517	7	0.6	100.0
**Total**	200 874	1113	100.0	

^a^The total population estimate and populations by age groups for 2019 are extrapolations from the 2016 census provided by the Samoa Bureau of Statistics.

^b^Total cases for which age data were available. Age was reported as “not stated” for 64 (5.4%) of the total 1177 cases recorded between 2008 and 2019.

**Figure 4.**
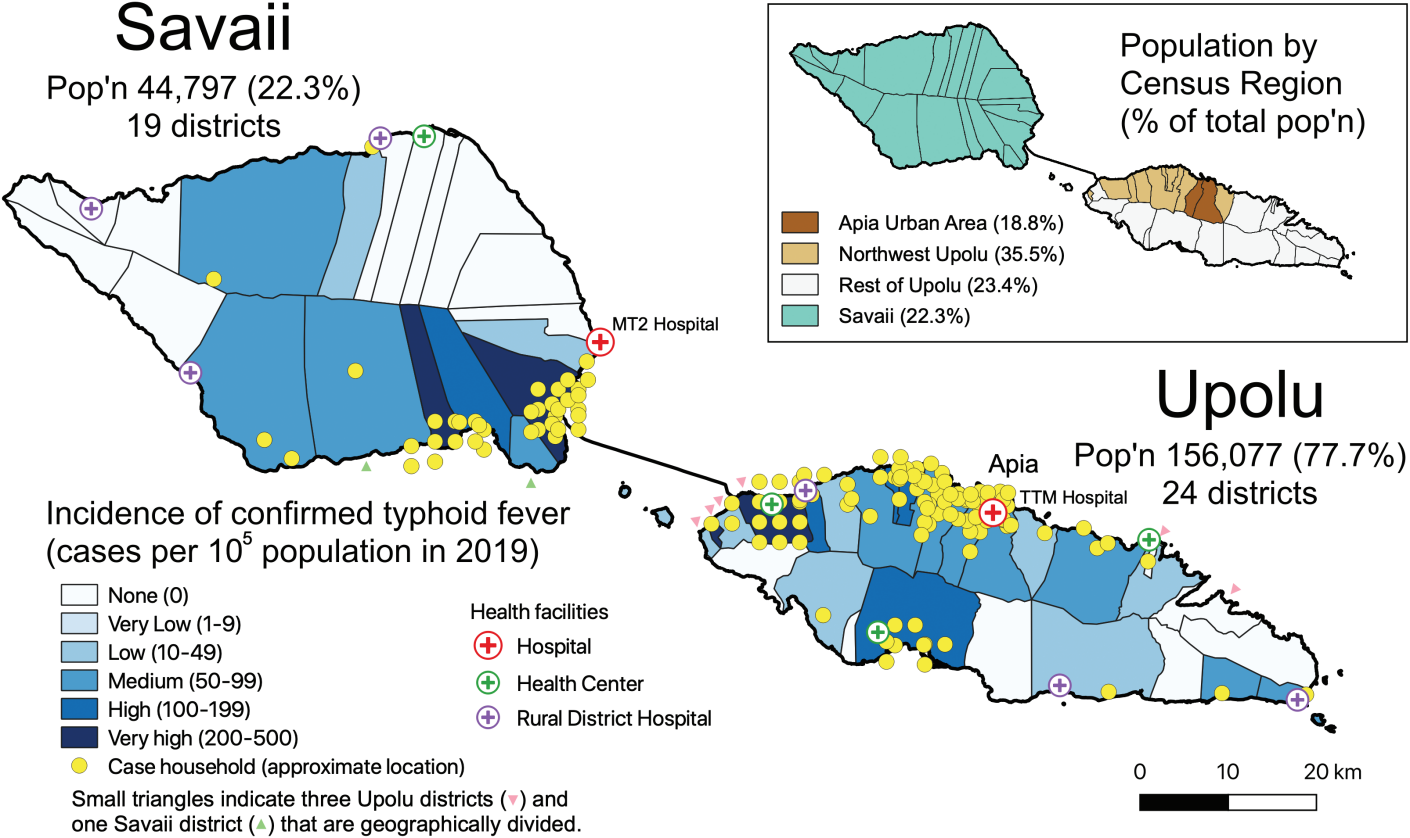
Map showing the incidence and geographical distribution of households of cases of blood culture-confirmed typhoid fever occurring in 2019 in Samoa. Incidence (cases per 10^5^ population) is represented by variable shading from white to dark blue, with darker hues representing higher incidence ranges. Each yellow dot approximates index case(s) of typhoid within a household to portray the density of cases. To maintain patient confidentiality, a mapping program randomly displaced the marker slightly on the map. Households of all cases were visited by a Samoa Typhoid Epidemiologic SWAT Team that recorded GPS coordinates with a Garmin GPSMAP 64SC. The insert shows the Apia Urban Area and 3 rural regions—Northwest Upolu, Rest of Upolu, and Savaii—with the percent of the 2019 population in parentheses. Upolu’s population of 156 077 comprises 77.7% of the total population of Samoa, whereas Savaii’s population of 44 797 comprises 22.3% of the total population. Mapping and point displacement were performed using QGIS 3.8.0-Zanzibar (QGIS Development Team, QGIS Geographic Information System, Open Source Geospatial Foundation Project, http://qgis.osgeo.org).

To account for the geographic distribution of typhoid cases in relation to population density, the proportion of total population by census region (Figure 4, insert), typhoid incidence by census region (Table 2), and typhoid incidence by district (for the 24 districts of Upolu and the 19 districts of Savaii) were calculated (Figure 4). On Upolu the incidence of typhoid was medium (50–99 cases/10^5^ population) or high (100–199 cases/10^5^ population) in districts along the Northwest Upolu coast. Unexpectedly, the incidence was found to be medium in the Apia Urban Area, where there is a reticulated water supply distributing putatively treated water. In contrast, except for 1 district on the mid-South coast, the remaining Upolu districts, which are primarily rural, showed a low incidence and sparse numbers of cases (Figure 4). Cases on Savaii mainly clustered along the populated areas near the ferry that travels to and from Upolu. Figure 4 also displays the case count burden of typhoid by geographic district. In this analysis, Northwest Upolu has the highest clustering of cases, particularly in districts along the coast, followed by the Apia Urban Area.

**Table 2.** Cases of Blood Culture-confirmed Typhoid Fever and Incidence (Cases/10^5^ Population) in 2019 by Census Region

Region	Population (2019 estimate)^a^	Total Cases (2019)	Incidence, Cases per 10^5^ pop.
**Apia Urban area**	37 785	23	60.9
**Northwest Upolu**	71 345	56	78.5
**Rest of Upolu**	46 947	20	42.6
**Savaii**	44 797	41	91.5
**Total**	200 874	140	69.7

^a^The total population estimate and populations by region for 2019 are extrapolations from the 2016 census provided by the Samoa Bureau of Statistics.

During the study period, Samoan *S*. Typhi strains remained susceptible to clinically relevant antimicrobials. All 140 isolates from 2019 were sensitive to ciprofloxacin, ampicillin, trimethoprim/sulfamethoxazole, ceftriaxone, and chloramphenicol. To place the pansensitive *S*. Typhi resistance patterns in context, we examined *Escherichia coli* and *Klebsiella* spp. (*K. pneumoniae*) blood culture isolates from 2019 at TTM Hospital for their antibiotic resistances. Of the 153 *E. coli* blood culture isolates (all ages), resistance was common to ciprofloxacin (11/47, 23.4%), ampicillin (98/153, 64.1%), trimethoprim/sulfamethoxazole (94/153, 61.4%), ceftriaxone (35/153, 22.9%), and chloramphenicol (3/21, 14.3%). Similarly, of the 47 *Klebsiella* isolates, resistance was common to ciprofloxacin (18/25, 72.0%), ampicillin (42/47, 89.4%), trimethoprim/sulfamethoxazole (22/47, 46.8%), ceftriaxone (25/47, 53.2%), and chloramphenicol (9/20, 45.0%).

## DISCUSSION

The review of blood culture-confirmed cases of typhoid from the period 2008 to 2019, which is already providing invaluable guidance to the nascent Samoa Typhoid Fever Control Program, includes data from: (i) 2008 through 2016, when blood culture capability and clinical microbiology were suboptimal; (ii) a transitional interval (2017 and the first half of 2018) when detection capacity was somewhat improved; and (iii) late 2018 and 2019 when the STFSI was fully operative and clinical microbiology was strong. The number of *S*. Typhi isolates increased markedly in 2019, likely reflecting improved detection capacity. Data prior to 2019 almost certainly represent a substantial underestimate of the true number of typhoid fever cases in Samoa. Similar strengthening of bacteriology practices for *Salmonella* detection in Fiji circa 2000 proved to be an investment that bore fruit in subsequent years when needed during typhoid outbreaks and epidemiologic investigations [17–20].

In Samoa the incidence of typhoid in infants and toddlers is low but increases in children 36–47 months of age. It elevates markedly in school age and peaks in adults, with little sex difference. The age-specific incidence pattern of typhoid in Samoa for the 5-year age groups 0–4 years through 40–44 years of age closely resembles the age pattern of endemic typhoid in Fiji [19], perhaps reflecting similar modes of transmission. Fiji typhoid overwhelmingly afflicts the indigenous Fijians, although sparing Indo-Fijians [17, 19, 20].

That the peak incidence of typhoid in Samoa occurred in young adults suggested that increased risk of exposure to *S*. Typhi might be occurring among adults when outside the household. Hypothesizing that males might be more likely to work outside the home and have greater exposure to *S*. Typhi, sex-specific incidence rates were compared for adults in the high-incidence age range of 25–39 years for the 7-year period of 2013–2019; the modestly higher incidence in males over females was insignificant.

During 2019, when *S.* Typhi detection was robust, district-specific incidence rates demonstrated several districts (4/24 on Upolu and 3/19 on Savaii) where the typhoid incidence was high or very high (≥100/10^5^) (Figure 4), as well as districts (6/24 on Upolu and 11/19 on Savaii) without cases; remaining districts exhibited intermediate typhoid incidences. This revelation of typhoid hotspots will help guide interventions. It would be useful to know the proportion of typhoid cases in Samoa that are due to short-cycle transmission from consumption of food vehicles contaminated by carriers (that might therefore contain large inocula), versus the proportion due to long-cycle transmission via ingestion of contaminated water (harboring smaller inocula). Regrettably, we do not yet have this information, although intensive prospective epidemiologic investigations are gathering information to help answer that question. Analysis of aggregated seasonality data may have precluded detection of insights on mechanism of transmission that might be gleaned if disaggregated date of onset could be combined with precise household GPS coordinates location, highly localized rainfall/hydrological data, and detailed epidemiologic data on contacts among confirmed cases. Those data are being collected prospectively since Q4 2018, and some of these data (e.g., GPS coordinates of case households) are being obtained by retrospective household visits. When sufficient data are in hand, they will be analyzed to try and discern patterns indicating short-cycle versus long-cycle transmission that are not presently evident from analyses of aggregated data.

Perusing the wealth of confirmed typhoid data, we derived several relevant insights. First, although there exist high-incidence hotspots (≥100 cases/10^5^), the overall typhoid incidence in Samoa was not high (<100/10^5^), nor was it so in any age group when 2008–2019 data were reviewed (Figure 3). Despite the spotty bacteriology capacity in early years, the data reveal a remarkable constancy of typhoid disease, year after year, with a similar incidence in school-age children and adults through age 39 years. Interestingly, the overall crude incidence of confirmed typhoid cases in Samoa in 2019, 69.7/10^5^ (Figure 1), closely resembles the incidence of 72/10^5^ used by Cvjetanović in his typhoid model that was based on Samoan data from the late 1960s [4]. These age-specific data are also invaluable for prioritizing targets for interventions.

In 2013 the Samoan government reaffirmed its commitment to tackle endemic typhoid. The 2013 roadmap for control of typhoid in Samoa was updated in 2017. The Samoa Typhoid Fever Control Program commenced in 2018 with an initial Preparatory Phase, the STFSI. This will be followed by an attack phase in which several interventions will be instituted, most importantly a mass vaccination of the high-risk population with Typbar-TCV™ (Bharat Biotech International Ltd, Hyderabad, India), the only WHO prequalified Vi conjugate vaccine [21–23]. A future consolidation phase that will follow the attack phase will include mop-up vaccinations and routine vaccination of toddlers 12 months of age when they receive measles vaccine through the Expanded Program on Immunization. The consolidation phase will also include an intensive nationwide search to detect chronic typhoid carriers, treat them with ciprofloxacin for 28 days [24] (local strains are susceptible) and monitor them with 3 (ideally nonconsecutive) stool cultures per month for up to 12 months posttherapy if they cease excreting, or every 2 months thereafter if they do not respond [24, 25].

Besides strengthening the clinical microbiology infrastructure, since late 2018 the STFSI has enhanced the Ministry of Health’s capacity to undertake intensive investigations of the epidemiology of endemic typhoid. Typhoid Epidemiologic “SWAT” Teams have been established, one for Upolu and the other for Savaii, that visit the households of all typhoid cases. The teams obtain detailed clinical, demographic, and risk-factor information from the index case and all household contacts, examine the contacts, and collect 3 rectal swabs from all contacts over several days. SWAT Teams are also trained to place Moore swabs [26, 27] to obtain environmental specimens for culture, when indicated. Two sentinel clinical/epidemiological surveillance sites were also established in rural areas, one on Upolu and another on Savaii, to obtain blood cultures from febrile patients (38^o^C for ≥ 3 days) suspected of having typhoid.

The attack phase will aim to achieve high coverage during a mass immunization campaign with the new WHO prequalified Vi conjugate vaccine, Typbar-TCV™. This vaccine elicits high seroconversion rates of serum immunoglobulin G antibodies in adults, children, toddlers, and infants as young as 6 months of age given a single parenteral dose and has shown 81.6% efficacy over 1 year of follow-up in a postlicensure randomized controlled field trial in Nepal [21, 23]. The fact that ~90% of all Samoan typhoid cases during 2008–2019 occurred in persons < 46 years of age, has led the nascent Samoa Typhoid Fever Control Program to target immunization of all Samoans 1–45 years of age with Typbar-TCV™. The expectation is for this vaccine to convert most susceptible Samoans postvaccination to a protected state in the face of continuing exposure to *S*. Typhi. Since immunization with Typbar-TCV™ diminishes the excretion of *S*. Typhi following ingestion of virulent typhoid bacilli [28], it is hoped that high vaccine coverage will reduce the circulation of *S*. Typhi across the islands and thereby progressively diminish the force of infection.

Although mass vaccination with Typbar-TCV™ is an attractive intervention that should yield a rapid impact if high coverage is achieved, we remain cognizant of the 1960s field trial of acetone-inactivated whole cell parenteral vaccine in Tonga [6] where that vaccine exhibited a lower level of protection than had been observed in several other WHO-sponsored field trials [7–10]. It was hypothesized that in Tonga food vehicles contaminated by carriers commonly contained large inocula of *S*. Typhi that could overwhelm the ability of the vaccine to protect some vaccinated subjects [6]. Volunteer challenge studies with virulent *S*. Typhi carried out by Hornick et al in the 1960s support this explanation [29]. They showed that with moderate challenge inocula the acetone-inactivated vaccine was the most protective killed whole cell vaccine tested [29]. However, when volunteers ingested a very high inoculum that caused a high attack rate in controls, the protective effect of the acetone-inactivated whole cell vaccine was overwhelmed [29].

In comparing the advantages in Samoa of vaccination as an intervention versus improving sanitation, Cvjetanović and colleagues concluded that both would be beneficial; however, if ≥1 additional vaccines of public health importance could be coadministered along with typhoid vaccine, the relative advantage of immunization increases [4, 5, 30]. The Samoa Typhoid Fever Control Program will coadminister measles vaccine with Typbar-TCV™ both during the mass immunization (to achieve a measles “catch-up” campaign in persons 1–21 years of age) and routinely in future years when 12-month-olds receive their measles/mumps/rubella vaccine. Collaborators from the Institute for Disease Modeling, who innovatively modeled data from the Chile Typhoid Fever Control Program of the 1980s [31], will use estimates of incidence, vaccine coverage, presumed proportion of short-cycle transmission, and other parameters to try and predict through modeling what will be the impact of the planned interventions.

The persisting moderate incidence of typhoid in Apia Urban Area, where the population is served by a reticulated, ostensibly treated, water supply, raises the question as to whether parts of the system supplying water to Apia may be faulty. Investigating this may identify an opportunity for a focused improvement of the water supply. The Fiji case-control study that investigated risk factors incriminated deficiencies of sanitation and water quality as significantly increasing risk of typhoid [20]. This may be true in Samoa as well, as Demographic and Health Survey and census surveys indicate widespread deficiencies of sanitation and of access to potable water [32].

Samoa’s relative isolation appears, heretofore, to have protected it from acquiring multiple antibiotic-resistant or extensively drug-resistant (XDR) *S*. Typhi. Thus, local strains respond readily to oral antimicrobials, if given expeditiously. Although antibiotics are only available through a physician’s signed prescription, antibiotic resistance is frequently observed in clinical blood culture isolates of *E. coli* and *Klebsiella* spp. in the clinical microbiology laboratories. The likely reason that these resistances, which are presumably encoded in part on plasmids, have not been acquired by Samoan *S*. Typhi is that most R factor plasmids are not of IncH1 incompatibility group. Most plasmid-borne AMR genes in *S*. Typhi reside on IncH1 plasmids. In the mid-1980s it was shown experimentally that R factor plasmids could be readily transferred from *E*. coli to *S*. Typhi, but they were not stably maintained [33]. The one exception is IncH1. Thus, the credible route for the emergence of drug resistant typhoid in Samoa is via importation of an *S*. Typhi strain from abroad. As Samoa expands as a tourist destination, and with increasing commerce with Fiji (where some endemic strains harbor AMR [34]), this threat of importation becomes increasingly relevant. Should an XDR strain arrive in Samoa and spread widely, current broad treatment options would diminish and would precipitate a public health crisis [35, 36].

Review of the existing burden data has provided insights into the epidemiology of typhoid in Samoa and has contributed to the design of both vaccine-based and water/sanitation-based interventions. These historical data, combined with enhanced microbiology and epidemiologic investigation capacities of the STFSI, provide a baseline against which the impact of the interventions can be measured. Indeed, it is high time to interrupt the tenacious, persisting endemicity of typhoid fever in Samoa.

## Notes

***Acknowledgments.*** Substantial assistance in data retrieval for the 2013 WHO consultancy was provided by Dr Tile Ah Leong-Lui in her role as a Public Health Physician for the Ministry of Health, Samoa.

***Financial support.*** This work was supported by Bill and Melinda Gates Foundation grant number OPP1194582 (PI: Prof. Myron M. Levine). The World Health Organization supported professor Levine’s consultation to Samoa in 2013. M. M. L. is supported in part by the Simon and Bessie Grollman Distinguished Professorship at the University of Maryland School of Medicine.

***Supplement sponsorship.* This supplement is funded with support from the Coalition against Typhoid Secretariat, housed at the Sabin Vaccine Institute in Washington, DC and made possible by a grant from the Bill & Melinda Gates Foundation.**

***Potential conflicts of interest.*** M. M. L. reports grants from Bill and Melinda Gates Foundation during the conduct of the study; in addition, M. M. L. has a patent entitled “Broad spectrum vaccine against typhoidal and nontyphoidal *Salmonella* disease” (US 9,011,871 B2) issued. R. M. R.-B. reports grants from Bill and Melinda Gates Foundation, grants from University of Maryland, and nonfinancial support from Government of Samoa, during the conduct of the study. S. M. T. reports grants from Bill and Melinda Gates Foundation during the conduct of the study; grants from Bill and Melinda Gates Foundation, outside the submitted work; in addition, S. M. T. has a patent titled “Broad spectrum vaccine against typhoidal and nontyphoidal *Salmonella* disease” (US 9,011,871 B2) issued. All other authors report no potential conflicts. All authors have submitted the ICMJE Form for Disclosure of Potential Conflicts of Interest. Conflicts that the editors consider relevant to the content of the manuscript have been disclosed.
